# A novel cytarabine crystalline lipid prodrug: Hexadecyloxypropyl cytarabine 3′,5′-cyclic monophosphate for proliferative vitreoretinopathy

**Published:** 2012-07-14

**Authors:** Jae Suk Kim, James R. Beadle, William R. Freeman, Karl Y. Hostetler, Kathrin Hartmann, Nadejda Valiaeva, Igor Kozak, Laura Conner, Julissa Trahan, Kathy A. Aldern, Lingyun Cheng

**Affiliations:** 1 Jacobs Retina Center at Shiley Eye Center, UC San Diego, La Jolla, CA; 2 Department of Medicine, San Diego VA Healthcare System and UC San Diego, La Jolla, CA; 3 Department of Ophthalmology, Sanggye Paik Hospital, Inje University, Seoul

## Abstract

**Purpose:**

The objectives of this study were to synthesize and characterize two types of cytarabine (Ara-C) lipid produgs and evaluate the prodrugs for sustained intraocular delivery after administration by intravitreal injection.

**Methods:**

Hexadecyloxypropyl cytarabine 5′-monophosphate (HDP-P-Ara-C) and hexadecyloxypropyl cytarabine 3′,5′-cyclic monophosphate (HDP-cP-Ara-C) were synthesized starting from cytarabine (1-β-D-arabinofuranosylcytosine). Their vitreal clearance profile was simulated using a custom dissolution chamber, in vitro cytotoxicity was evaluated using cell proliferation assays, and in vivo ocular properties in rat and rabbit eyes were assessed using biomicroscopy, indirect ophthalmoscopy, tonometry, electroretinography, and histology.

**Results:**

HDP-P-Ara-C was cleared from the dissolution chamber (flow rate 2 µL/min) within 7 days. In contrast, HDP-cP-Ara-C, a much more insoluble prodrug, was still detectable 36 days after the dissolution process was started. HDP-P-Ara-C had a 50% cytotoxicity concentration of 52±2.6 μM in human retinal pigment epithelium (ARPE-19) and 32±2.2 µM in a rat Müller cell line, rMC-1. The 50% cytotoxicity concentration values for HDP-cP-Ara-C in ARPE-19 and rMC-1 cells were 50 µM and 25 µM, respectively. HDP-P-Ara-C was not detectable 2 weeks after the highest intravitreal dose (228 µg/rat eye) was injected, and no ocular toxicity was found. With HDP-cP-Ara-C, the drug depot was visible for 26 weeks following a single intravitreal injection (800 µg/rabbit eye). For both compounds, the electroretinogram, intraocular pressure, and other toxicity studies were negative except for the highest dose of HDP-cP-Ara-C (800 µg/eye), which had focal toxicity from the direct touch of the retina and decreased dark adapted a-waves and decreased flicker electroretinogram amplitudes (generalized estimating equations, p=0.039 and 0.01).

**Conclusions:**

The cyclic monophosphate prodrug, HDP-cP-Ara-C, was found to have physiochemical properties better suited for sustained delivery of cytarabine to posterior segments of the eye. These properties included limited aqueous solubility, in vitro antiproliferative activity, and good tolerability after injection into rabbit eyes.

## Introduction

Proliferative vitreoretinopathy (PVR) is a pathological, exuberant scarring process that is the most common cause of failure after rhegmatogenous retinal detachment surgery [1-3]. The technical difficulty of surgical repair, as well as the fact that anatomic success does not necessarily lead to functional improvement, has led to a search for less invasive and more effective methods for preventing and treating PVR [4-7].

The underlying causes of PVR are complex; however, a major component appears to be cell proliferation on the surface and undersurface of the retina after damage to the blood–retinal barrier exposes retinal cells to vitreal growth factors and cytokines [2]. Due to the excessive fibrocellular proliferation present in PVR, several treatment strategies have investigated the use of antiproliferative agents such as daunorubicin or 5-fluorouracil [5-7]. However, another important consideration in developing drugs for preventing PVR is that it typically occurs approximately 6 weeks after the initial retina reattachment surgery. Ideally, anti-PVR pharmacologic treatment should provide therapeutic levels of drug in the eye for several months without adding additional ocular or systemic side effects.

Our efforts to develop long-lasting agents for preventing PVR have focused on synthesizing and evaluating sparingly soluble prodrugs of antiproliferative nucleoside analogs that could be administered by intravitreal injection. For example, we recently described hexadecyloxypropyl 5-fluoro-2'-deoxyuridine cyclic 3′,5′-monophosphate, a lipid prodrug of 5-fluoro-2′-deoxyuridine that stays in the vitreous cavity for months after intravitreal injection and inhibited experimental PVR, presumably by continually releasing minute amounts of the prodrug from its crystalline form into the vitreous [8].

Cytarabine (1-β-D-arabinofuranosylcytosine, Ara-C) is another pyrimidine analog that impairs cellular proliferation by inhibiting DNA synthesis. Cytarabine demonstrated better in vitro antiproliferative efficacy on retinal pigment epithelial and fibroblast cells than 5-fluorouracil (5-FU) [9]. However, like other polar nucleoside analogs, local treatment using unmodified cytarabine requires frequent postoperative injections due to rapid clearance from the vitreous. To continue our efforts to design an effective local treatment for PVR, we synthesized two lipid prodrugs of cytarabine, hexadecyloxypropyl cytarabine 5′-monophosphate (HDP-P-Ara-C) and hexadecyloxypropyl cytarabine cyclic 3′,5′-monophosphate (HDP-cP-Ara-C), and evaluated their antiproliferative activity and release kinetics in vitro and their ocular properties in rat and rabbit eyes.

## Methods

### Chemistry

#### General

All reagents were of commercial quality and used without further purification unless indicated otherwise. Chromatographic purification was done using the flash method with silica gel 60 (230–400 mesh; EMD Chemicals, Inc., Gibbstown, NJ). Proton nuclear magnetic resonance (^1^H NMR) spectra were recorded on Varian HG spectrophotometers operating at 400 MHz and are reported in units of parts per million (ppm) relative to internal tetramethylsilane at 0.00 ppm. Electrospray ionization mass spectra (ESI-MS) were recorded on a Finnigan LCQDECA spectrometer in positive or negative mode. Purity of the compounds was also assessed with thin layer chromatography (TLC) using Analtech silica gel-GF (250 μm) plates and the solvent system: CHCl_3_/MeOH/con NH_4_OH/H_2_O (70:30:3:3 v/v). TLC results were visualized with ultraviolet light, phospray (Supelco, Bellefonte, PA), and charring at 400 °C. Cytarabine (cytosine arabinoside, Ara-C) was purchased from Aldrich Chemical Co (Milwauke, WI).

### Synthesis of hexadecyloxypropyl cytarabine 5′-monophosphate (HDP-P-Ara-C)

To a solution of hexadecyloxypropyl-3-phosphate (545 mg, 1.4 mmol, prepared by treating 3-(hexadecyloxy)-1-propanol with phosphorous oxychloride as described by Ruiz et al. [10]) and 1-β-D-arabinofuranosyl cytosine (350 mg, 1.4 mmol) in dry pyridine (30 ml) was added *N*,*N*-dicyclohexylcarbodiimide (577 mg, 2.8 mmol). The mixture was stirred for 3 days at room temperature. Then the mixture was filtered, and the solvent evaporated. The residue was adsorbed on silica gel and purified with flash column chromatography. Gradient elution CH_2_Cl_2_ to 20% EtOH/CH_2_Cl_2_ gave HDP-P-Ara-C as a white powder. Yield: 325 mg (38%). ^1^H NMR (CDCl_3_/methanol-*d_4_*): δ 7.78 (d, 1H); 6.10 (d, 1H); 5.85 (d, 1H); 3.99–3.87 (m, 2H); 3.50–3.25 (m, 2H); 3.95–4.05 (m, 2H); 3.46 (t, 2H); 3.37 (t, 3H); 1.80 (pentet, 2H); 1.50–1.47 (m, 2H); 1.27–1.24 (m, 26H); 0.88 (t, 3H). ESI-MS (*m/z*) 604 [M-H]^-^.

### Synthesis of hexdecyloxypropyl cytarabine 3′,5′-cyclic monophosphate (HDP-cP-Ara-C)

#### N^4^, 2´-O-dibenzoyl-3′,5′-(tetraisopropyl disiloxane-1,3-diyl)cytosine arabinoside (1)

Benzoyl chloride (15.7 ml, 0.14 mol) was added dropwise at 0 °C to a solution of 3′,5′-O-(tetraisopropyl disiloxane-1,3-diyl) cytosine arabinoside (9.37 g, 19.0 mmol, prepared from cytarabine and 1,3-dichloro-1,1,3,3-tetraisopropyldisiloxane as described by Markiewicz et al. [11]) and 4-dimethylaminopyridine (0.21 g, 1.9 mmol) in 200 ml of anhydrous pyridine. The mixture was left for 30 min at room temperature and then was stirred for 5 h at 70 °C. The suspension was cooled to 0 °C, and the reaction was quenched by adding 70 ml of water. Volatiles were removed, and the residue was coevaporated with toluene. The crude product was purified with column chromatography on silica gel. Eluent: ethyl acetate/hexanes (80:20), then ethyl acetate. Yield: 10.5 g (80%). ^1^H NMR (CDCl_3_/methanol-*d_4_*): δ 8.22 (d, 1H, *J*=7 Hz); 8.07 (d, 2H, *J*=7 Hz); 7.99 (d, 2H, *J*=7 Hz); 7.25–7.84 (m, 6H); 6.46 (d, 1H, *J*=6 Hz); 6.03 (t, 1H, *J*=7Hz); 4.45–4.65 (m, 1H); 3.90–4.23 (m, 5H); 0.8–1.20 (m, 24H). ESI-MS (*m/z*) 694.14 [M+H]^+^, 716.21 [M+Na]^+^.

#### N^4^, 2´-O-dibenzoyl-cytosine arabinoside (2)

Acetic acid (2.9 g) and ammonium fluoride (8.1 g) were added to a mixture of N^4^, 2´-O-dibenzoyl-3′,5′-tetraisopropyl disiloxane-1,3-diyl)cytosine arabinoside (10.5 g, 15.13 mmol) in dry methanol (250 ml). The mixture was stirred at room temperature overnight before the solvents were evaporated under vacuum. The residue was purified by column chromatography on silica gel. Eluent: dichloromethane: from 5% to 15% of methanol. Yield: 6.3 g (92%). ^1^H NMR (CDCl_3_/methanol-*d_4_*): δ 8.49 (d, 1H, *J*=7 Hz); 7.97 (d, 2H, *J*=7 Hz); 7.82 (d, 2H, *J*=7 Hz); 7.58–7.68 (m, 2H); 7.43–7.58 (m, 2H); 7.33–7.43 (m, 2H); 6.45 (d, 1H, *J*=6 Hz); 5.70 (t, 1H, *J*=7Hz); 4.40–4.42 (m, 1H); 4.12–4.16 (m, 1H); 3.85–3.96 (m, 2H); 3.34–3.36 (m, 1H). ESI-MS (*m/z*) 452.01 [M+H]^+^, 474.09 [M+Na]^+^.

#### N^4^, 2´,5′-O-tribenzoyl-cytosine arabinoside (3)

A solution of benzoyl chloride (1.62 ml, 14.00 mmol) in anhydrous pyridine (25 ml) was added dropwise to a suspension of N^4^, 2’-O-dibenzoyl-cytosine arabinoside (6.3 g, 13.96 mmol) in anhydrous pyridine (100 ml) at 0 °C. The reaction mixture was stirred for 3 h at 0 °C and then quenched by adding water (50 ml). The mixture was concentrated under vacuum, the residue was dissolved in CHCl_3_ (200 ml) and washed with water (2×20 ml), and the CHCl_3_ fraction was evaporated. The residue was purified by column chromatography on silica gel. Eluent: ethyl acetate/hexanes (80:20), then ethyl acetate. Yield: 5.4 g (69%). ^1^H NMR (CDCl_3_/methanol-*d_4_*): δ 8.27 (d, 1H, *J*=8 Hz); 8.05 (d, 2H, *J*=8 Hz); 7.96 (d, 2H, *J*=8 Hz); 7.81(d, 2H, *J*=8 Hz); 7.37–7.66 (m, 9H); 6.49 (d, 1H, *J*=5 Hz); 5.68 (t, 1H, *J*=7Hz); 4.76–4.85 (m, 1H); 4.63–4.70 (m, 1H); 4.45–4.50 (m, 2H); 4.08–4.15 (m, 1H). ESI-MS (*m/z*) 555.93 [M+H]^+^, 578.06 [M+Na]^+^.

#### Hexadecyloxypropyl N^4^,2´,5′-O-tribenzoyl-cytosine arabinoside, 3′-monophosphate (4)

To a solution of N^4^, 2′,5′-tribenzoyl cytosine arabinoside (2.0 g, 3.6 mmol) and hexadecyloxypropyl phosphate (2.74 g, 7.2 mmol) in dry N,N-dimethylformamide (50 ml), *N*,*N*-dicyclohexylcarbodiimide (1.5 g, 7.2 mmol) was added. The mixture was stirred for 50 h at 90 °C. The solvent was evaporated, and then the residue was purified by column chromatography on silica gel. Eluent: CH_2_Cl_2_ with from 5% to 15% MeOH. Yield: 2.2 g (67%). ^1^H NMR (CDCl_3_/methanol-*d_4_*): δ 8.27 (d, 1H, *J*=8 Hz); 8.07 (d, 2H, *J*=7 Hz); 7.93 (d, 2H, *J*=7 Hz); 7.83 (d, 2H, *J*=7 Hz); 7.48–7.61 (m, 6H); 7.35–7.42 (m, 3H); 6.48 (d, 1H, *J*=6 Hz); 5.90 (t, 1H, *J*=7Hz); 4.83–4.95 (m, 1H); 4.72–4.78 (m, 1H); 4.02–4.08 (m, 1H); 3.91–3.97 (m, 2H); 3.49–3.55 (m, 2H); 3.33–3.44 (m, 4H); 1.66–1.75 (m, 2H); 1.46–1.63 (m, 2H); 1.21–1.39 (m, 26H); 0.89 (t, 3H, *J*=7Hz). ESI-MS (*m/z*) 916.32 [M-H]^-^.

### Hexadecyloxypropyl cytosine arabinoside, 3′-monophosphate

Hexadecyloxypropyl N^4^, 2´,5′-O-tribenzoyl-cytosine arabinoside, 3′-monophosphate (2.2 g, 2.4 mmol) was dissolved in dry pyridine (10 ml), and then MeOH/NH_3_ (7 N solution) (25 ml) was added. The mixture was stirred overnight at room temperature before the solvent was evaporated. The residue was purified by column chromatography on silica gel. Eluent: CH_2_Cl_2_: from 5% to 15% of methanol. Yield: 0.66 g (46%). ^1^H NMR (CDCl_3_/methanol-*d_4_*): δ 7.90 (d, 1H, *J*=8 Hz); 6.19 (d, 1H, *J*=6 Hz); 5.88 (t, 1H, *J*=7Hz); 4.49–4.51 (m, 1H); 4.40–4.42 (m, 1H); 4.13–4.18 (m, 1H); 3.95–4.05 (m, 2H); 3.85–3.95 (m, 2H); 5.56 (t, 1H, *J*=7 Hz); 3.43 (t, 1H, *J*=7 Hz); 1.89–1.95 (m, 2H); 1.54–1.59 (m, 2H); 1.21–1.35 (m, 26H); 0.89 (t, 3H, *J*=7 Hz). ESI-MS (*m/z*) 606.11 [M+H]^+^, 628.22 [M+Na]^+^, 644.16 [M+K]^+^.

### Hexadecyloxypropyl cytosine arabinoside, 3′,5′-cyclic-monophosphate

A solution of hexadecyloxypropyl cytosine arabinoside 3′-monophosphate (0.64 g, 1.05 mmol) in dry pyridine (250 ml) was added dropwise to a solution of triisopropylbenzenesulfonyl chloride (3.2 g, 10.5 mmol) and 5-methyl-imidazole (17.0 g, 1.4 ml) in dry pyridine (300 ml). The mixture was stirred for 2 days at room temperature. Solvents were evaporated under vacuum, and the residue was purified by column chromatography on silica gel. Eluent: ethyl acetate/[chloroform:methanol:NH_4_OH:water 80:20:1:1] (100/75%). Yield: 80 mg of equatorial isomer, 80 mg of axial isomer, and 80 mg of mixture these two isomers; total: 240 mg (39%). ^1^H NMR (CDCl_3_/methanol-*d_4_*): δ 7.36 (d, 1H, *J*=8 Hz); 6.36 (d, 1H, *J*=6 Hz); 5.74 (t, 1H, *J*=7Hz); 4.69–4.75 (m, 1H); 4.50–4.55 (m, 1H); 4.20–4.26 (m, 1H); 3.95–4.05 (m, 2H); 3.85–3.95 (m, 2H); 3.35–3.60 (m, 4H); 1.69–1.72 (m, 2H); 1.54–1.59 (m, 2H); 1.21–1.35 (m, 26H); 0.89 (t, 3H, *J*=7 Hz). ESI-MS (*m/z*) 588.22 [M+H]^+^, 620.04 [M+Na]^+^.

### Simulated intravitreal clearance kinetics

**Figure 1:**
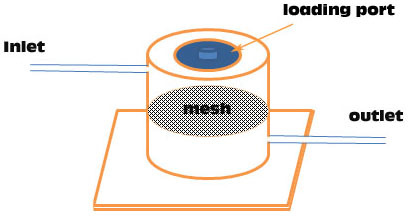
The dissolution chamber is made of poly methyl methacrylate. Inlet tubing connects from a syringe pump to the upper part of the chamber, and the outlet tubing leaves from the lower part of the chamber to the sample collector. In the center of the dissolution chamber, a titanium mesh circle is installed to prevent the large drug particles reaching the lower chamber to possibly clog the outgoing tubing. The mesh contains 5-µM pores.

Dissolution starting from an intravitreal drug depot was simulated using a custom chamber and a syringe pump. The drug powder (600 µg) was weighed into the chamber, which had 1.5 ml of effective circulation volume to mimic the rabbit vitreous. The chamber was filled with 1.5 ml of Hank’s Balanced Salt Solution (HBSS). The chamber was then connected by tubing to a NE-1000 syringe pump (New Era Pump Systems, Farmingdale, NY) on one end and open tubing on the other end so that the solution could be collected into a vial (Figure 1). The syringe pump was set to a flow rate of 2 µl/min [12], and the syringe filled with HBSS. The entire setup was maintained at 37 °C and pumped continuously. This dissolution chamber mimics the rabbit vitreous fluid turnover only. The chamber does not mimic vitreal physiologic situations, which so far cannot be reproduced in vitro. At the same time every day, the vial was changed and the contents collected and stored at −80 °C until liquid chromatography/mass spectrometry (LC/MS) was performed.

### Biology

#### Cell lines

Human retinal pigment epithelium (ARPE-19) cells were obtained from the American Type Culture Collection (ATCC; Manassas, VA). A rat Müller cell line, rMC-1, was provided by Dr. Gabe Silva at UC San Diego (originally obtained from Dr. Vijay Sarthy, Northwestern University, Chicago, IL). ARPE-19 cells were cultured in Dulbecco’s modified Eagle medium (DMEM)/F12 and rMC-1 in DMEM. DMEM/F12 medium and DMEM were maintained at 37 °C in a humidified chamber of 5% CO_2_. All culture media were supplemented with 10% fetal bovine serum, 100 U/ml penicillin, and 100 µg/ml streptomycin. The cell lines were chosen due to their dominant presence in the pathology of proliferative vitreoretinopathy [13,14].

#### Cell proliferation assay (rMC-1/XTT)

rMC-1 cells were plated in 96-well plates at 1×10^−4^ cells per well. Serial dilutions of the compounds were added to the wells in a total volume of 100 µl. The plates were maintained in a 37 °C incubator. After 5 days, 50 µl of a solution of XTT (Roche Molecular Biochemicals, Indianapolis, IN) was added to each well, and the plates returned to the incubator for 30 min, after which the ocular density (OD) of each well was measured at a wavelength of 450 nanometers. Percent inhibition was determined by comparing the OD of wells containing compound to the OD of control wells.

#### Cell proliferation assay (ARPE-19/WST-1)

ARPE-19 cells were plated in 96-well plates at 1×10^−4^ cells per well. Serial dilutions of the compounds were added to the wells in a total volume of 100 µl. The plates were maintained in a 37 °C incubator. After 5 days, 10 µl of a solution of WST-1 (Roche Diagnostics, Indianapolis, IN) was added to each well, and the plates returned to the incubator for 0.5 h, after which the OD_450_ of each well was measured. Percent inhibition data were obtained from at least three experiments with at least six wells at each concentration in separate 96-well plates. The mean optical density values corresponding to the untreated controls were taken as 100%. The results were expressed as the percentage of the optical density of treated cells relative to that of untreated controls.

### In vivo studies in animal eyes

All animal studies were conducted in adherence with the ARVO Statement for the Use of Animals in Ophthalmic and Vision Research.

#### Toxicity study of hexadecyloxypropyl cytarabine 5′-monophosphate in rat eyes

Twelve Brown Norway rats were used to evaluate the effect of intravitreal HDP-P-Ara-C versus control injection. HDP-P-Ara-C was intravitreally injected resulting in concentrations of 0.06 mM (2 µg/eye), 0.112 mM (4 µg/eye), 1.12 mM (40 µg/eye), 2.0 mM (73 µg/eye), and 6.32 mM (228 µg/eye) in rat vitreous. Only one eye of each rat was injected with the drug, and the fellow eye was injected with saline as control. Electroretinograms (ERGs) were obtained before drug injection and 1 day before scheduled sacrifice. Following injection, toxicity and safety were evaluated with indirect ophthalmoscopy, biomicroscopy, and fundus photography. At week 2 following drug injections, all of the rats were euthanized with intraperitoneal injection of ketamine (100 mg/kg) and xylazine (10 mg/kg) followed by intraperitoneal injection of 120 mg/kg sodium pentobarbital. Six eyes were used for pathologic evaluation and six eyes used for evaluation of vitreous drug level. Vitreous drug levels were determined at the end of the experiment using high-performance liquid chromatography (HPLC).

#### Toxicity study of hexadecyloxypropyl cytarabine cyclic 3′,5′-monophosphate in rabbit eyes

Sixteen New Zealand Red rabbits were used for this study. Only one eye of each animal was used for the drug testing, and the fellow eye was used as a control by injection of the diluent. Four doses were tested, and each dose was tested in four rabbit eyes after a 50 µl intravitreal injection of the HDP-cP-Ara-C suspension with a resulting dose of 25 µg/eye, 80 µg/eye, 250 µg/eye, and 800 µg/eye. After the intravitreal injection, the rabbits were examined at week 1, 2, 4, 8, and 12 and then once a month for 7 months. The eyes were monitored with slit-lamp exam, tonometry, indirect ophthalmoscope, and fundus camera. The drug depot size was estimated and recorded using the optic disc area and the drug depot contour in the vitreous. The number of disc areas for estimating the two dimensions of the drug depot and the contour (ball=3, patch=2, filaments and dots=1) provides an estimate of the density of the drug depot [8]. ERGs were recorded from all animals at week 2, week 8, and immediately before the animals were euthanized by deeply anesthetizing the animals with a combined ketamine (24 mg/kg) and xylazine (6 mg/kg) intramuscular injection followed by an intravenous injection of 0.3 ml/kg B-euthanasia (Schering-Plough Animal Health, Kenilworth, NJ) [8]. After the animals were euthanized, the eye globes were fixed in 2% paraformaldehyde and 1.5% glutaraldehyde overnight and processed for paraffin embedding and light microscopy.

### Statistical analysis

Intraocular pressure (IOP) measurements and ERG recordings were repeated measurement data. Therefore, generalized estimating equations were used to compare the mean IOP and ERG amplitudes between each treatment group and the control group [15,16]. All analyses were performed with SAS 9.2 software (SAS Institute Inc., Cary, NC), and the difference was considered to be significant when the p value was smaller than 0.05.

## Results

### Chemistry

**Figure 2:**
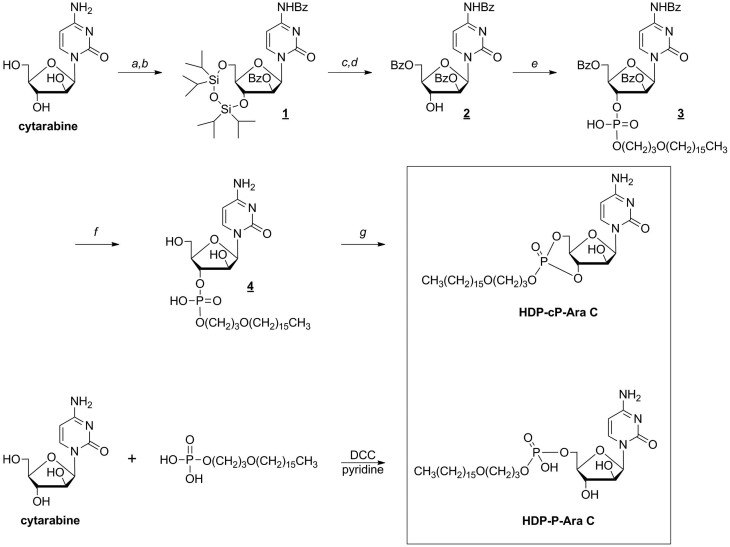
Synthesis of hexadecyloxypropyl cytarabine 5’-monophosphate and hexadecyloxypropyl cytarabine 3’,5’-cyclic monophosphate. The following reagents were used: a) 1,3-dichloro-1,1,3,3-tetraisopropyldisiloxane, pyridine; b) benzoyl chloride, pyridine; c) tetrabutylammonium fluoride, HOAc, CH_3_CN; d) benzoyl chloride, pyridine, e) hexadecyloxypropyl phosphate, *N*,*N*-dicyclohexylcarbodiimide, pyridine; f) ammonia/methanol; g) 2,4,6-triisopropylbenzenesulfonyl chloride, pyridine.

The two lipid prodrugs of cytarabine were successfully synthesized starting from cytarabine. The synthesis procedure is summarized in Figure 2, and detailed procedures and characterization data are provided in the Methods section.

### Drug effects on cell proliferation and viability

**Figure 3:**
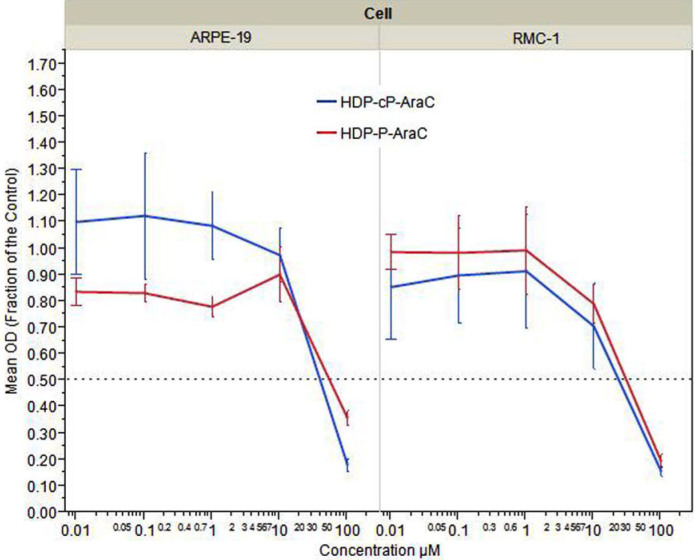
Dose response curve showing the effect of hexadecyloxypropyl cytarabine 3’,5’-cyclic monophosphate (HDP-cP-Ara-C) and hexadecyloxypropyl cytarabine 5’-monophosphate (HDP-P-Ara-C)on human retinal pigment epithelium (ARPE-19) cells and rat Müller (rMC-1) cells proliferation. The y-axis represents the fraction of mean optical density (OD) values of testing wells for the compounds by the mean OD values of the cells for medium control using WST-1 or XTT cell proliferation assay.

The effect on cell proliferation of HDP-P-Ara-C and HDP-cP-Ara-C was tested in two ocular cell lines, ARPE-19 and rMC-1. The CC_50_ values were 52 µM in ARPE-19 and 32 µM in rMC-1 for HDP-P-Ara-C, and 50 µM and 25 µM for HDP-cP-Ara-C, respectively (Figure 3). Both compounds were more potent in inhibiting rat Müller cells (rMC-1) than ARPE-19 cells.

### Simulated intravitreal clearance kinetics

**Figure 4:**
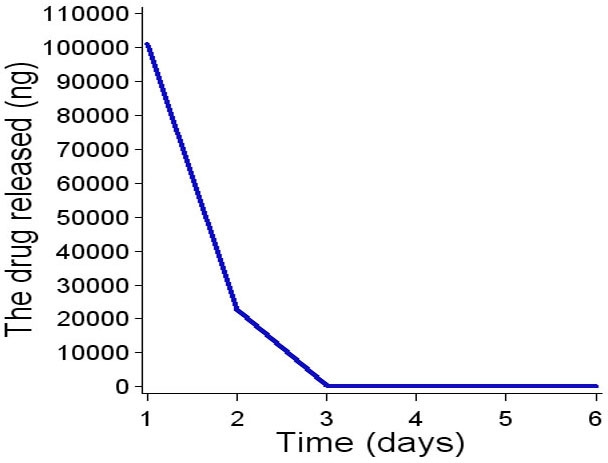
In vitro hexadecyloxypropyl cytarabine 5’-monophosphate (HDP-P-Ara-C) release profile. HDP-P-Ara-C completely released from the dissolution chamber within 6 days.

**Figure 5:**
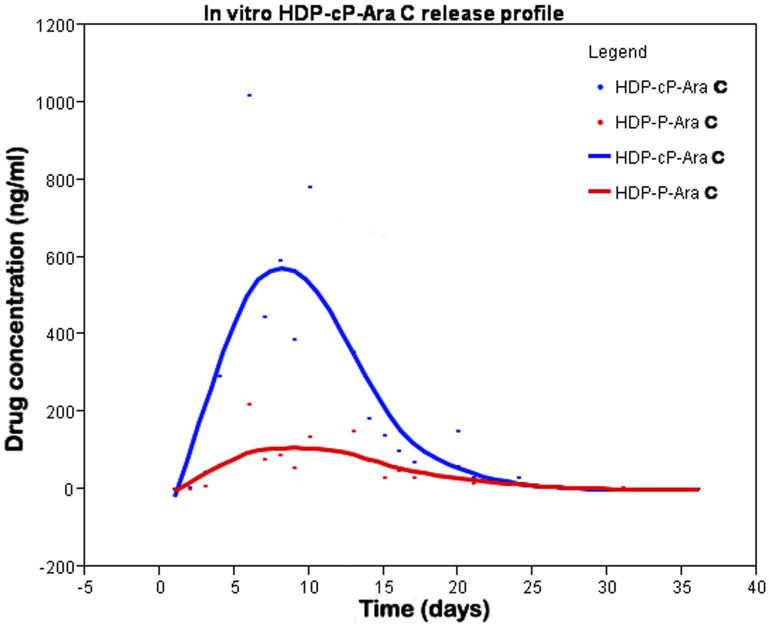
In vitro hexadecyloxypropyl cytarabine 3’,5’-cyclic monophosphate (HDP-cP-Ara-C) release profile. HDP-cP-Ara-C had a peak release around day 10, and the compound was still detectable at the end of the experiment (day 36). HDP-cP-Ara-C and hexadecyloxypropyl cytarabine 5’-monophosphate (HDP-P-Ara-C) were detected in the dissolution chamber eluate.

HDP-P-Ara-C in vitro release showed a fast washout. Within 3 days, 99% of the drug was cleared from the chamber (Figure 4). On day 7 and after, no drug was detected in the samples. In contrast, clearance of HDP-cP-Ara-C from the chamber demonstrated a bell-shaped release with a long tail on the right side (Figure 5). HDP-cP-Ara-C and HDP-P-Ara-C were detected in the eluate samples, indicating that some of the release was due to hydrolysis of the cyclic monophosphate to give the more soluble prodrug HDP-P-Ara-C. Release of both compounds increased from the start to day 9, decreased from day 9 to 21, and then leveled off until the end of the experiment. The amount of HDP-P-Ara-C released was much lower than the cyclic analog at each point.

### Hexadecyloxypropyl cytarabine 5′-monophosphate in rat eyes

No toxicity was found with any dose injected. After injection, the compound did not form a depot in the vitreous, and the vitreous remained clear. ERG and pathology revealed normal retinal physiology and histology (data not shown). HPLC analysis revealed that no drug was detectable at week 2 following intravitreal injection (including the eyes with the highest dose).

### Hexadecyloxypropyl cytarabine cyclic 3′,5′-monophosphate in rabbit eyes

**Figure 6:**
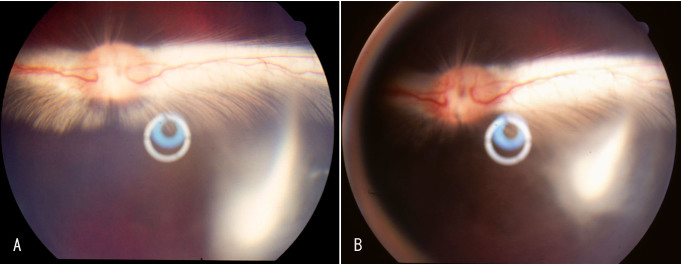
Color fundus photograph of an experimental eye showing hexadecyloxypropyl cytarabine 3’,5’-cyclic monophosphate (HDP-cP-Ara-C) drug depot at 16 weeks (**A**) and at 28 weeks (**B**) after the intravitreal injection of 800 μg of crystals. Panel **B** shows about 40% to 50% decrease of the drug depot over 12 weeks.

**Figure 7:**
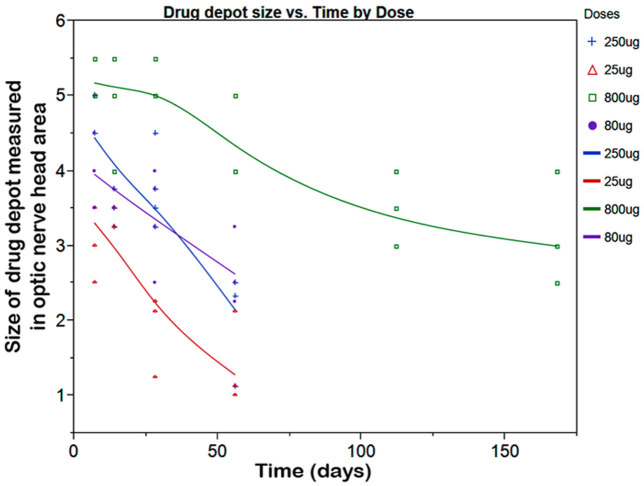
This graph illustrates the change in hexadecyloxypropyl cytarabine 3’,5’-cyclic monophosphate (HDP-cP-Ara-C) intravitreal drug depot size over time. The drug depot size was measured in the unit of optic nerve disc area by an indirect ophthalmoscope at each checking point. The drug depots from all the doses were visible 2 months after the initial intravitreal injection. The drug depot of the highest dose was still visible 7 months before the animals were euthanized.

**Figure 8:**
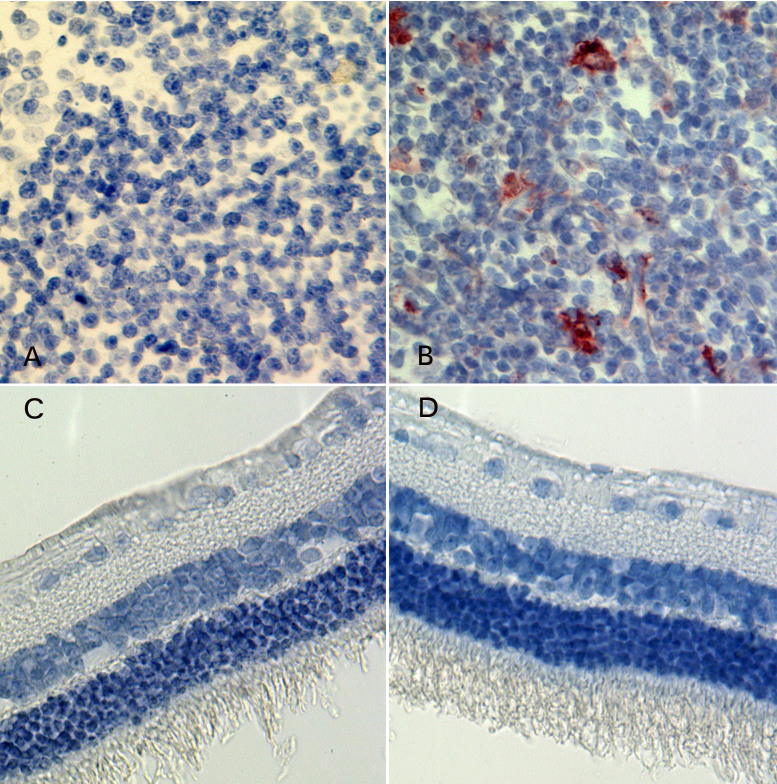
Light microscopic graphs of the retina and spleen. **A** and **B** are from the spleen tissue. The **A** serves as a negative control, and the **B** serves as a positive control for the terminal deoxynucleotidyl transferase dUTP nick end labeling (TUNEL) assay for the retina. **D** is from study eye that received an intravitreal injection of 250 μg of hexadecyloxypropyl cytarabine 3’,5’-cyclic monophosphate (HDP-cP-Ara-C), and frame **C** is from the fellow control eye of the same rabbit. Both retinas show normal structures and negative apoptosis staining (82.5X).

In the in vitro study, we noted that HDP-cP-Ara-C was minimally water soluble. We reasoned that the drug depot formed in the vitreous would interfere with fundus exams if we used rat eyes that have a very large lens and very limited vitreous cavity. Rabbit eyes were a better choice for evaluating this formulation. During the 7-month study, two eyes out of the four eyes injected with the 800-µg dose showed direct contact between the drug depot and the optic nerve or inferior retina causing medullary ray gliosis and discoloration of the inferior retina. No other toxicity was noted with the other eyes injected with the 800-µg dose or lower doses. The vitreous drug depot experienced a very slow decrease in size (Figure 6). The depot was visible before the scheduled euthanasia in all eyes and showed a decrease over time (Figure 7). Table 1 summarizes the clinical observations. The intraocular pressure analysis showed no significant difference between the injected eyes and the fellow eyes. The ERG examination did not reveal drug toxicity in the eyes that received 250-µg or lower doses. However, the dark adapted a-wave and the amplitude of flicker ERGs from the eyes of the 800-µg HDP-cP-Ara-C were significantly lower than their fellow control eyes (Table 2). Terminal deoxynucleotidyl transferase dUTP nick end labeling staining was performed on the paraffin sections from the eyes that received the 250-µg intravitreal injections. No structural abnormality or apoptosis was revealed when compared to the sections from the counter-lateral eyes that did not receive an intravitreal injection and served as an internal control (Figure 8). On the same sections for the apoptosis staining, the lens was normal, and no apoptosis was observed (data not shown).

**Table 1:** Summary of clinical observations after injection of HDP-cP-Ara-C in rabbit eyes.

**Time (weeks)**	**Dose (µg)**	**AC cell**	**Vit. Clarity**	**Fundus abnormality**
1	25	0/4	1/4#	0/4
1	80	0/4	0/4	0/4
1	250	0/4	0/4	0/4
1	800	0/4	0/4	0/4
8	25	0/4	0/4	0/4
8	80	0/4	0/4	0/4
8	250	0/4	0/4	0/4
28	800	0/4	0/4	2/4*

Clinical observation revealed no toxicity for intravitreal 250 µg or lower doses of HDP-cP-Ara-C. However, out of 4 eyes with intravitreal 800 µg of HDP-cP-Ara-C, two eyes had retinal toxicity. Footnote: #-indicating the vitreous haze came from vitreous hemorrhage during the drug injection *-one eye showed direct contact between drug depot and the medullary ray and the other showed direct contact between drug depot and the inferior retina.

**Table 2:** Summary of ERG examination sata.

**Group**	**Dark A amp**	**p value**	**Dark B amp**	**p value**	**Light A amp**	**p value**	**Light B amp**	**p value**	**Flicker amp**	**p value**
low OD	−14.44	p=0.72	88.14	p=0.5	−4.49	p=0.18	47.93	p=0.5	22.58	p=0.8
ctrl OS	−15.4		83.76		−3.92		46.92		21.56	
mid OD	15.32	p=0.98	79.31	p=0.73	−5.64	p=0.44	42.83	p=0.42	23.51	p=0.68
ctrl OS	−15.3		76.44		−4.49		45.41		24.15	
high OD	−15.6	p=0.64	83.19	p=0.71	−3.57	p=0.67	49.68	p=0.06	25.07	p=0.47
ctrl OS	−16.47		84.75		−3.76		47.52		25.64	
HH OD	−11.69	p=0.039	95.35	p=0.18	−5.57	p=0.74	48.06	p=0.23	21.09	p=0.01
ctrl OS	−15.49		100.86		−5.43		51.79		27.82	

ERG examinations revealed no ERG abnormality with the eyes injected with 250 µg of HDP-cP-Ara-C or lower doses. However, the eyes with 800 µg of HDP-cP-Ara-C demonstrated lower dark adapted a-wave and lower amplitude of flicker ERG. Each dose was tested in four eyes of four rabbits, and their fellow eyes were used as controls. So n=4 for each dose group and n=4 for its corresponding control group. low=low dose (25 µg/eye); mid=moderate dose (80 µg/eye); high=high dose (250 µg/eye); HH=the highest dose (800 µg/eye); ctrl=control. amp=amplitude; dark=dark adapted ERG; light=light adapted ERG.

## Discussion

Cytarabine is a Food and Drug Administration–approved nucleoside analog used as a chemotherapy agent for hematologic malignancies such as lymphoma, including intraocular lymphoma [17-19]. In addition, cytarabine has been studied as a local ocular application for inhibiting ocular proliferation [20-22]. To be effective for intraocular usage, a drug must possess sufficient intravitreal half-life; otherwise, frequent injections are needed. Unmodified nucleosides such as cytarabine typically have an intravitreal half-life of 12 h or less, and even liposome-encapsulated nucleosides have a vitreous half-life of only a few days [21]. For trauma or retinal surgery–related PVR prevention or treatment, a slow release system that could keep cytarabine levels above the therapeutic level in the vitreous for 2 to 4 months would be ideal because PVR forms and exacerbates during this period [23].

Our approach to sustained PVR therapy involves the intravitreal application of sparingly soluble alkoxyalkyl esters of antiproliferative nucleoside phosphates (or phosphonates) [24]. After intravitreal injection, crystalline forms of these prodrugs persist in the vitreous cavity for a long time while releasing minute amounts of drug. For example, we previously described hexadecyloxypropyl cyclic cidofovir, a potent antiviral agent that can suppress herpes simplex virus for up to 4 months after a single intravitreal injection [25]. We hypothesized that similar prodrugs of antiproliferative nucleosides might inhibit ocular cell proliferation, and recently reported preliminary studies using the hexadecyloxypropyl esters of 9-[2-phosphonomethoxyethyl]guanine [26] and 2’-deoxy-5-fluorouridine 3’,5’-cyclic monophosphate [27].

In the present study, we synthesized two prodrugs of cytarabine, HDP-P-Ara-C and HDP-cP-Ara-C. Cytotoxicity studies in vitro indicated that both compounds possess more potent antiproliferative activity than unmodified Ara-C, consistent with several earlier studies that show alkoxyalkyl ester prodrugs increase cell membrane permeability and lead to higher intracellular concentrations of active antiviral or antiproliferative metabolites [27,28]. Notably, HDP-cP-Ara-C had a CC_50_ value of 25 µM in rMC-1 cells, which was slightly more potent than that previously reported for a similar prodrug, HDP-P-Ara-G (a mean CC_50_ value of 41 µM in the same cell line), that produced significant inhibition of a laser-induced rat choroidal neovascularization model in a prophylactic study design [27].

Our simulation of intravitreal clearance using the non-cyclic prodrug HDP-P-Ara-C showed that it was cleared rapidly from the release chamber with all of the drug released by day 6. Compounds of this type are analogs of phospholipids and readily form micelles in water, which probably accounts for the rapid clearance. This behavior was also confirmed in the in vivo study in rat eyes in which no HDP-P-Ara-C was detected in the vitreous 2 weeks after the injection.

Since micellization of HDP-P-Ara-C led to rapid clearance, we also evaluated the corresponding cyclic analog HDP-cP-Ara-C. Cyclic monophosphates lack a negatively charged head group and are therefore non-micelle forming and sparingly soluble in water. After HDP-cP-Ara-C was introduced into the release chamber, daily HPLC analysis of the eluate showed a gradual increase in the free drug level. This peaked around 10 days and then decreased thereafter. Low-level sustained release was detected from day 25 to the last time point (day 36) with the drug concentration at about 16 ng/mL in the eluate. This release profile was similar to our previous in vivo rabbit vitreous aspirate study in which a similar formulation of hexadecyloxypropyl ganciclovir monophosphate was studied [25].

Interestingly, in the simulated intravitreal clearance study of HDP-cP-Ara-C, HDP-cP-Ara-C and HDP-P-Ara-C were detected in the eluate with HPLC analysis, indicating that slow hydrolysis of HDP-cP-Ara-C to the more soluble form HDP-P-Ara-C was occurring in PBS during the experiment. These clearance studies are meant to simulate the turnover of water in living rabbit eyes. Thus, the observed release kinetics are relevant to the behavior of drug in a living eye. Both forms of the drug accumulate in retinal tissue [29] and are metabolized to the active metabolite Ara-C triphosphate, which inhibits cell division after its misincorporation into nascent DNA.

In the in vivo study, the HDP-cP-Ara-C drug depots at various doses were observed at the time of euthanasia, and estimation of the vitreal clearance of the drug indicated that the drug depot would be visible for 3 months after 80 or 250 µg/eye doses and even longer with the higher doses.

### Conclusions

Hexadecyloxypropyl cytarabine 5’-monophosphate was synthesized from cytarabine and found to be unsuitable for providing slow release after intravitreal injection. However, the corresponding 3’, 5’-cyclic monophosphate analog was sparingly water soluble, active against proliferating ocular cells, and thus appeared suitable for evaluation as a long-lasting intravitreal treatment. The in vivo safety of HDP-cP-Ara-C was evaluated, and doses up to 250 µg/eye were well tolerated. In addition to HDP-cP-Ara-C, which possesses a hexadecyloxy propyl ester, we hypothesize that varying the length of the alkoxyalkyl moiety to optimize the water solubility/clearance rate of this compound might improve its efficacy. We recognize that cataract is a possible complication of systemic use of cytarabine and its derivatives. Therefore, in future studies, lens toxicity needs to be closely monitored upon adjusting the length of the alkoxyalkyl moiety to optimize water solubility of the compound. In addition, a pharmacokinetic study of cytarabine delivery to the retina is imperative for optimizing this compound for preventing and treating PVR.
